# Phosphorylation of endogenous α-synuclein induced by extracellular seeds initiates at the pre-synaptic region and spreads to the cell body

**DOI:** 10.1038/s41598-022-04780-4

**Published:** 2022-01-21

**Authors:** Shiori Awa, Genjiro Suzuki, Masami Masuda-Suzukake, Takashi Nonaka, Minoru Saito, Masato Hasegawa

**Affiliations:** 1 Department of Brain and Neurosciences, Tokyo Metropolitan Institute of Medical Science, Tokyo, Japan; 2 Department of Biosciences, College of Humanities and Sciences, Nihon University, Tokyo, Japan; 3 Department of Correlative Study in Physics and Chemistry, Graduate School of Integrated Basic Sciences, Nihon University, Tokyo, Japan; 4 Present Address: Graduate School of Frontier Biosciences, Osaka University, Osaka, Japan

**Keywords:** Cell biology, Neuroscience

## Abstract

Accumulation of phosphorylated α-synuclein aggregates has been implicated in several diseases, such as Parkinson's disease (PD) and dementia with Lewy bodies (DLB), and is thought to spread in a prion-like manner. Elucidating the mechanisms of prion-like transmission of α-synuclein is important for the development of therapies for these diseases, but little is known about the details. Here, we injected α-synuclein fibrils into the brains of wild-type mice and examined the early phase of the induction of phosphorylated α-synuclein accumulation. We found that phosphorylated α-synuclein appeared within a few days after the intracerebral injection. It was observed initially in presynaptic regions and subsequently extended its localization to axons and cell bodies.　These results suggest that extracellular α-synuclein fibrils are taken up into the presynaptic region and seed-dependently convert the endogenous normal α-synuclein that is abundant there to an abnormal phosphorylated form, which is then transported through the axon to the cell body.

## Introduction

In neurodegenerative diseases such as Alzheimer's disease, Parkinson's disease (PD) and amyotrophic lateral sclerosis, the neurodegeneration is thought to be caused by the accumulation of abnormal protein aggregates characteristic of each disease^1,2^. For example, abnormal α-synuclein protein aggregates are accumulated in neuronal and/or glial cells in PD, dementia with Lewy bodies (DLB) and multiple system atrophy (MSA), and therefore these diseases are termed α-synucleinopathies^3^. In PD and DLB, accumulations of phosphorylated α-synuclein aggregates are mainly observed in neurons in the form of Lewy bodies (LBs) and Lewy neurites (LNs), while they are seen in oligodendrocytes as glial cytoplasmic inclusions (GCIs) in MSA. The abnormal α-synuclein deposits observed in the brains of individuals are accumulated as fibrous or filamentous forms with cross-β structures, existing in phosphorylated and partially ubiquitinated states^4^. These abnormal α-synuclein species exhibit seeding activity for prion-like conversion, being similar in this respect to the infectious forms of prion protein causing Creutzfeldt–Jakob disease and bovine spongiform encephalopathy. Moreover, these abnormal aggregates can propagate throughout the brain in a prion-like manner^1,5^. Various other neurodegenerative disease-related proteins, including amyloid-β, tau and TDP-43, are also thought to propagate through neural networks in a similar manner.

α-Synuclein is a natively unfolded protein of 140 amino acid residues, which is abundant in the brain, and is normally found in both soluble and membrane-associated fractions^3^. In the brain, α-synuclein is mainly localized in presynaptic termini of neurons^3,6^. Although its physiological function has not been fully clarified, it appears to be involved in the regulation of synaptic vesicle recycling and neurotransmitter release^7–10^. In addition, α-synuclein is also thought to be involved in the regulation of dopamine, a type of neurotransmitter that is critical for the pathogenesis of PD. In familial forms of α-synucleinopathies, missense mutations and multiplication of the *SNCA* gene encoding α-synuclein have been reported, indicating that structural changes or excessive amounts of α-synuclein protein are involved in the development of α-synucleinopathies^11^.

Recombinant soluble α-synuclein proteins form amyloid-like fibrils that are morphologically and physicochemically similar to those observed in patients’ brains. These synthetic α-synuclein fibrils can act as seeds and induce seeded aggregation of α-synuclein by converting normal monomers into abnormal aggregates^12–16^. Introduction of synthetic α-synuclein fibrils into cultured cells or primary-cultured neurons leads the accumulation of phosphorylated α-synuclein aggregates in the cells. Intracerebral injection of synthetic α-synuclein fibrils also induces phosphorylated and ubiquitinated α-synuclein pathologies even in wild-type (WT) mice. It has also been reported that extracts from the brains of individuals with α-synucleinopathies induce α-synuclein pathologies in cellular and animal models^13,17–20^. When these α-synuclein aggregates were injected into one side of the brain, phosphorylated α-synuclein pathology also appeared on the contralateral side, and the pathology was concentrated at specific locations dependent upon the injection site^21^. Moreover, the propagation of the pathology was dependent on synaptic activity^22^. These findings suggest that α-synuclein aggregates may spread through neural circuits.

The internalization of α-synuclein seeds by neural or glial cells is a key step in the prion-like propagation of α-synuclein aggregates, but so far little is known about the uptake mechanism. Several mechanisms have been proposed for the uptake of α-synuclein aggregates. One is the lymphocyte-activation gene 3 (LAG3)-mediated uptake mechanism^23^. LAG3 is an α-synuclein fibril-binding protein. Deletion of LAG3 or application of antibodies against LAG3 reduces the internalization of α-synuclein fibrils and the induction of the accumulation of α-synuclein aggregates in primary neurons. Deletion of LAG3 also attenuates the induction of α-synuclein pathology in mice injected with α-synuclein fibrils. However, another group recently reported that LAG3 is not expressed in neurons and the role of LAG3 in the spreading of α-synuclein pathology is not valid^24^. Another mechanism for the uptake of α-synuclein fibrils is heparin sulfate proteoglycan-mediated macropinocytosis^25^. It was also reported that α-synuclein fibrils are trafficked along the endolysosomal pathway in primary neurons^26,27^.

Previous studies in WT mouse models injected with α-synuclein fibrils have demonstrated the spread of α-synuclein aggregates throughout the brain when observed after a long period of time, such as several months^12,13,28^. However, much remains unclear about how the injected α-synuclein fibrils induce aggregation and accumulation of intrinsic α-synuclein. We also know only a little about when and where endogenous α-synuclein undergoes modifications such as phosphorylation and ubiquitination. In this study, we injected WT mice with α-synuclein fibrils, and observed the changes in intrinsic α-synuclein over time, focusing on the early period after α-synuclein fibril injection. We examined when α-synuclein fibrils are taken up by the neurons and how intrinsic α-synuclein aggregates are formed.

## Results

**Figure 1:**
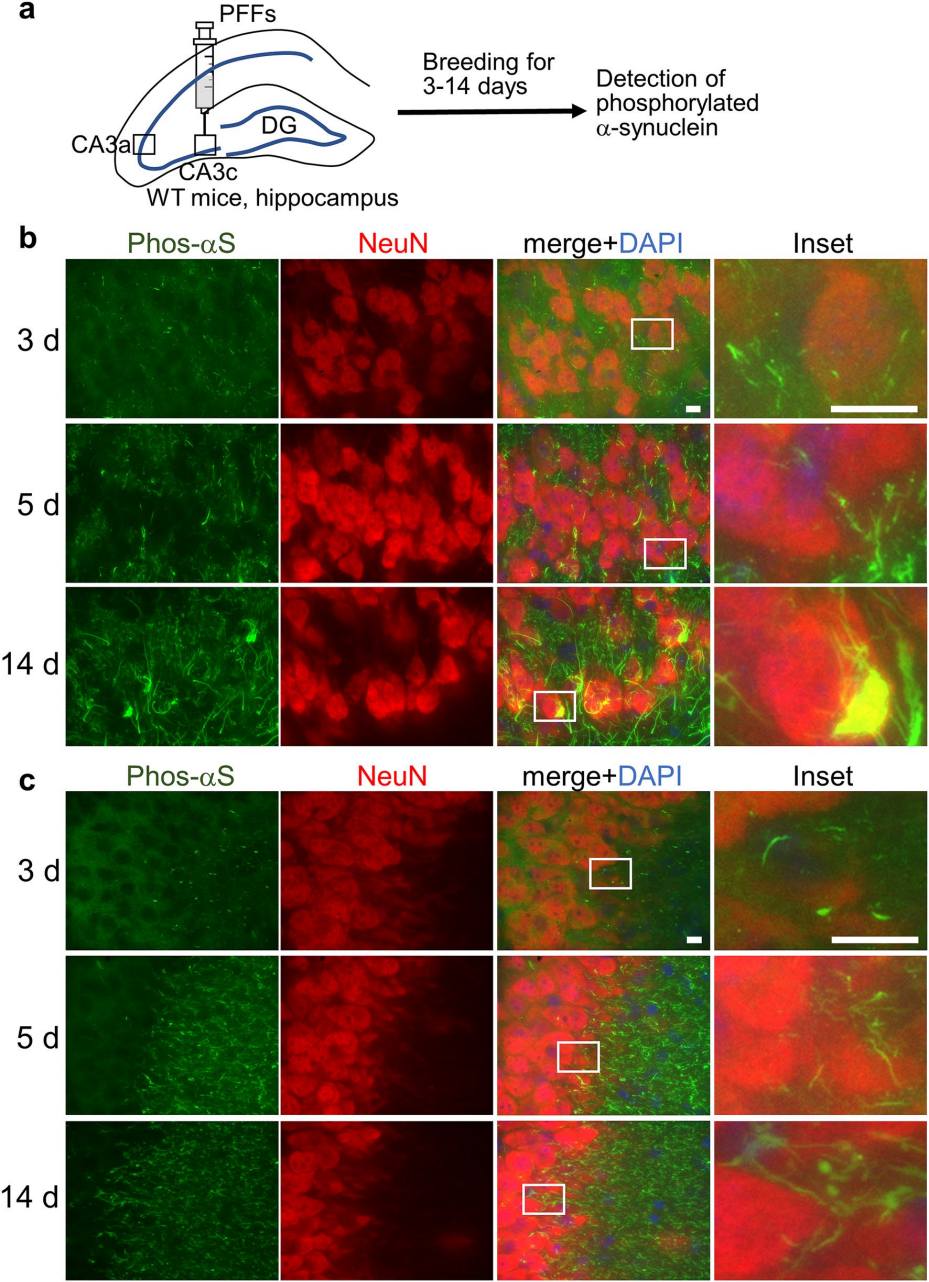
Seed-dependent phosphorylation of α-synuclein in mouse brain. (**a**) Schematic representation of seed-dependent phosphorylation of α-synuclein after the injection into mouse brain. α-Synuclein PFFs were injected into the CA3c region of mouse hippocampus. Mice were sacrificed after 1 to 14 days, and the brains were fixed and stained with anti-phosphorylated α-synuclein. (**b**) Appearance of phosphorylated α-synuclein (Phos-αS) in the CA3c region at 3 days (upper), 5 days (middle) or 14 days (lower) after the injection. Images show phosphorylated α-synuclein (green), NeuN (red), a neuronal cell body marker, DAPI (blue) and the merged image in the CA3c region. Regions surrounded by white rectangles in merged images are magnified and shown in the rightmost panel of each lane. (**c**) Appearance of phosphorylated α-synuclein in the CA3a region at 3 days (upper), 5 days (middle) or 14 days (lower) after the injection. Images show phosphorylated α-synuclein (green), NeuN (red), DAPI (blue) and the merged image in the CA3a region. Regions surrounded by white rectangles in merged images are magnified and shown in the rightmost panel of each lane. Scale bars, 10 μm.

To examine how neuronal cells take up α-synuclein fibrils and how the fibrils induce endogenous α-synuclein aggregates, we injected α-synuclein pre-formed fibrils (PFFs) into mouse brains and followed the induction of seed-dependent aggregation of α-synuclein. First, we prepared the fibrils to be injected. The S129A mutant mouse α-synuclein, in which serine 129 of mouse α-synuclein is replaced with alanine, was produced in *E. coli* and purified^29^. Fibril formation was induced by agitating the monomer at 37 °C for 1 week. The resulting PFFs were injected into the hippocampal CA3c region of WT mice on one side (Fig. 1a). The reason for injecting into the hippocampus is that ordered neuronal layers are formed in the hippocampus, and we wanted to observe fibril uptake and seed-dependent aggregate formation, rather than prion-like propagation^30^. In addition, we observed high expression of α-synuclein in the hippocampal presynaptic region, in line with previous findings (Supplementary Fig. 1)^31,32^. To observe early seed-dependent phosphorylation by fibril uptake, we injected higher amounts of PFFs into the mouse brain than most previous reports^12,13,28^. To monitor seed-dependent aggregate formation by injected PFFs, we used antibodies that recognize α-synuclein phosphorylated at serine 129. Since the PFFs we used for injection have serine 129 replaced by alanine, it is considered that the antibodies do not recognize the injected α-synuclein PFFs, but only recognize the phosphorylation of endogenous α-synuclein^29^. While the accumulation and propagation of phosphorylated α-synuclein have been observed several months after the injection in previous intracerebral injection experiments^12,13,33^, we focused here on when and where the uptake and the seed-dependent aggregation of α-synuclein take place by observing the appearance of phosphorylated α-synuclein in the first few days after the injection. We examined the appearance of phosphorylated α-synuclein at 3, 5, and 14 days after the intracerebral injection and found that the appearance of dots of phosphorylated α-synuclein had already begun after 3 days (Fig. 1b, c). Further phosphorylated α-synuclein in the form of dots, projections, and aggregates appeared over time. Co-localization with the neuronal marker NeuN revealed that phosphorylated α-synuclein accumulated in neurons. We also examined the appearance of phosphorylated α-synuclein at 3, 5, and 14 days after the intracerebral injection of WT α-synuclein PFFs and obtained similar results (Supplementary Fig. 2). In addition, we confirmed that no phosphorylated α-synuclein was induced by the injection of α-synuclein monomers even at 14 days after the injection (Supplementary Fig. 3). We also examined the appearance of phosphorylated α-synuclein at one day after the injection and found that α-synuclein had not been phosphorylated at this point (Supplementary Fig. 4).

**Figure 2:**
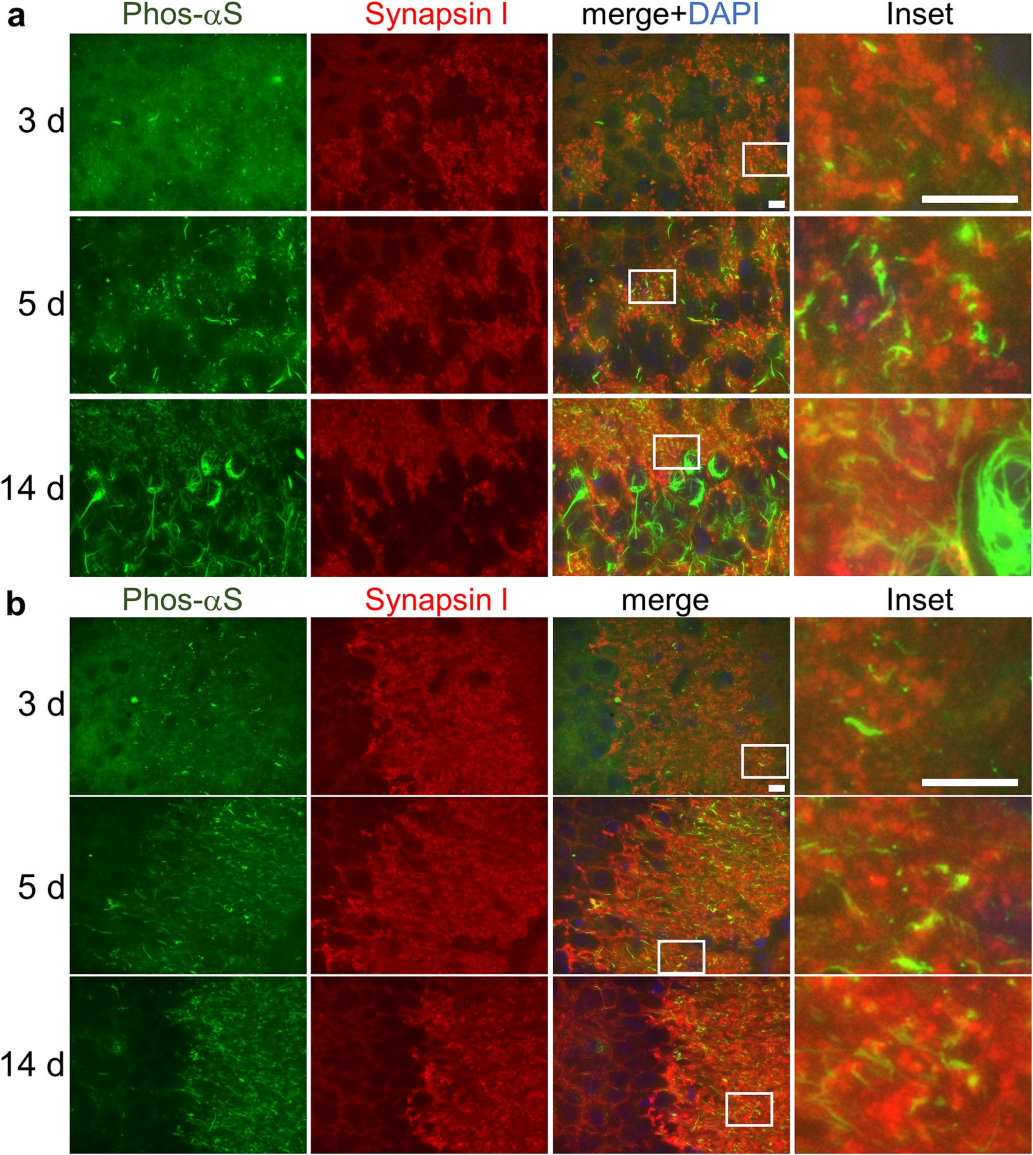
Seed-dependent phosphorylation of α-synuclein in pre-synaptic regions of mouse neurons. (**a**) Appearance of phosphorylated α-synuclein (Phos-αS) in the CA3c region at 3 days (upper), 5 days (middle) or 14 days (lower) after the injection. Images show phosphorylated α-synuclein (green), synapsin I (red), a presynaptic region marker, DAPI (blue) and the merged image in the CA3c region. Regions surrounded by white rectangles in merged images are magnified and shown in the rightmost panel of each lane. (**b**) Appearance of phosphorylated α-synuclein in the CA3a region at 3 days (upper), 5 days (middle) or 14 days (lower) after the injection. Images show phosphorylated α-synuclein (green), synapsin I (red), DAPI (blue) and the merged image in the CA3a region. Regions surrounded by white rectangles in merged images are magnified and shown in the rightmost panel of each lane. Scale bars, 10 μm.

**Figure 3:**
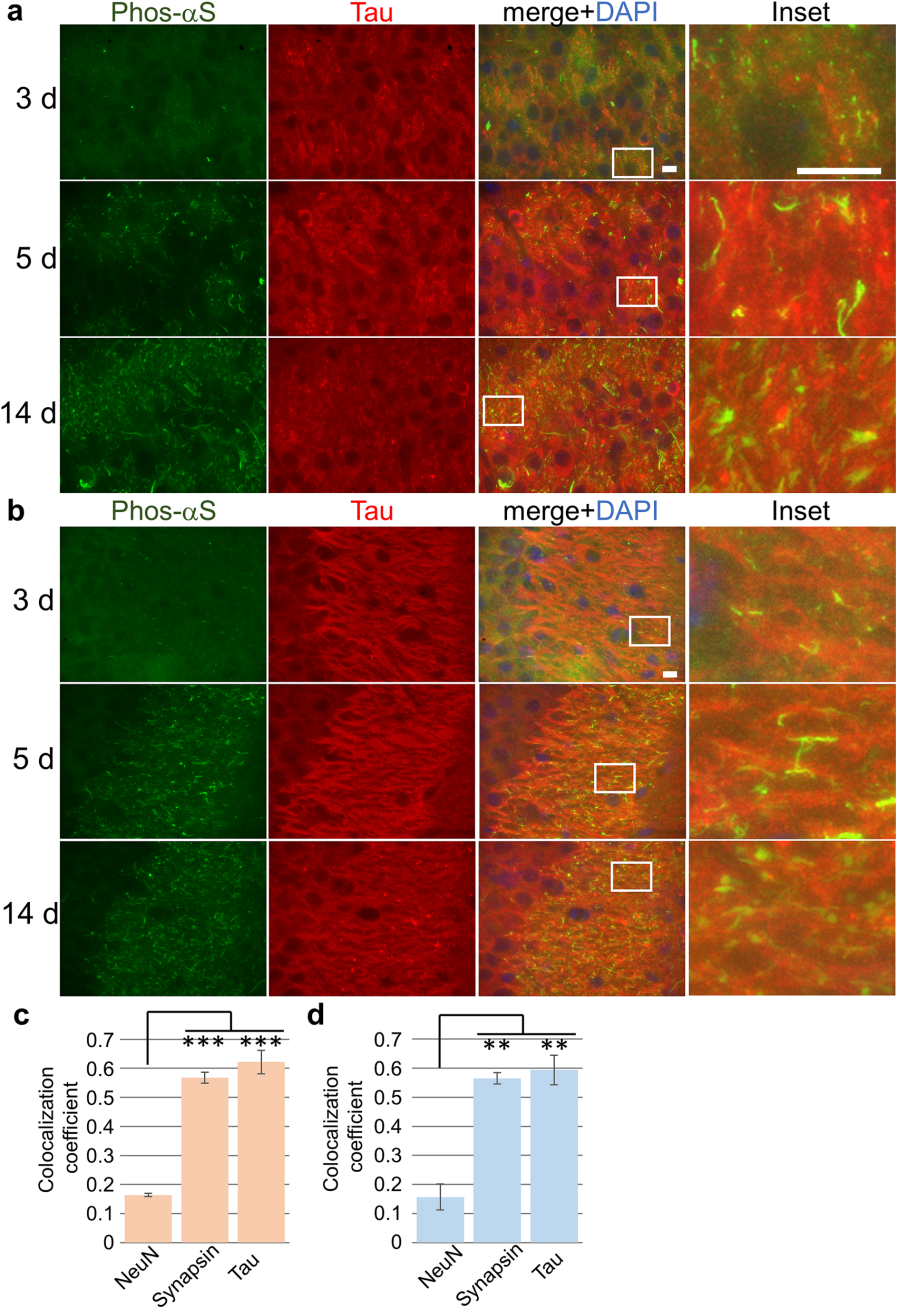
Seed-dependent phosphorylation of α-synuclein in axons of mouse neurons. (**a**) Appearance of phosphorylated α-synuclein (Phos-αS) in the CA3c region at 3 days (upper), 5 days (middle) or 14 days (lower) after the injection. Images show phosphorylated α-synuclein (green), tau (red), an axon marker, DAPI (blue) and the merged image in the CA3c region. Regions surrounded by white rectangles in merged images are magnified and shown in the rightmost panel of each lane. (**b**) Appearance of phosphorylated α-synuclein in the CA3a region at 3 days (upper), 5 days (middle) or 14 days (lower) after the injection. Images show phosphorylated α-synuclein (green), tau (red), DAPI (blue) and the merged image in the CA3a region. Regions surrounded by white rectangles in merged images are magnified and shown in the rightmost panel of each lane. (**c**) and (**d**) Quantification of the colocalization coefficient between phosphorylated α-synuclein and indicated markers at 5 days after the injection in the CA3c region (**c**) and the CA3a region (**d**) (mean ± S.E.M; n = 3). Statistical analysis was performed using one-way ANOVA and Tukey post hoc test. **P < 0.01, ***P < 0.001. Scale bars, 10 μm.

α-Synuclein has been reported to be abundant in the presynaptic region^3^. Therefore, to investigate whether the dot-shaped phosphorylated α-synuclein is localized at the presynaptic region, we examined its co-localization with the presynaptic marker synapsin I and found that it is indeed co-localized with synapsin I (Fig. 2). This indicates that the phosphorylated α-synuclein found early after the intracerebral injection, such as 3 days, is located in presynaptic regions. This in turn suggests that the injected α-synuclein fibrils taken up into these regions converts the endogenous α-synuclein that is abundant there to the abnormal phosphorylated form. Next, we examined the co-localization of tau, an axonal marker, and phosphorylated α-synuclein in the brain of injected mice and found that some projections of phosphorylated α-synuclein co-localized with tau more than 5 days after the injection (Fig. 3a, b). To verify the colocalization between phosphorylated α-synuclein and these neuronal markers, we quantified the co-localization coefficients between these markers at 5 days after the injection (Figs. 1, 2, 3) and confirmed that synapsin I and tau co-localized with the phosphorylated α-synuclein at 5 days after the injection while NeuN did not (Fig. 3c, d). Moreover, co-localizations of phosphorylated α-synuclein with MAP2 or NF-L, other neurite markers, were examined, and it was found that some projections of phosphorylated α-synuclein also co-localized with MAP2 and NF-L (Supplementary Fig. 5). These results indicate that phosphorylated α-synuclein is localized in the axon. In the mouse brain injected with α-synuclein fibrils, phosphorylated α-synuclein, which looks to form aggregates, appeared in the cell body 14 days after the injection (Fig. 1b). We stained these phosphorylated α-synuclein with FSB which bound to b-sheet rich amyloid like aggregates^34^. We found that the most of aggregate-like phosphorylated α-synuclein and some of process-like phosphorylated α-synuclein were stained with FSB while the most of dot-like structures were not (Supplementary Fig. 6). Thus, we thought that some of phosphorylated α-synuclein observed at 14 days after the injection were amyloid-like fibrillary aggregates. Accumulations of α-synuclein aggregates are also seen in oligodendrocytes as GCIs in the brain of MSA patients^3^. Thus, we examined whether phosphorylated α-synuclein appearances are also seen in glial cells. We examined co-localization of Iba1, GFAP or CNPase and phosphorylated α-synuclein at 14 days after the injection and found that the accumulations of phosphorylated α-synuclein rarely colocalized at all with these glial cell markers (Supplementary Fig. 7). Thus, the phosphorylated α-synuclein that appeared soon after seed injection, such as within 14 days, was present mainly in neurons. This might be due to the high neuronal expression of endogenous α-synuclein, which may be important for prion-like propagation. We also examined whether these phosphorylated α-synuclein was ubiquitinated and confirmed that the most of the phosphorylated α-synuclein was ubiquitinated as seen in the brain of patients (Supplementary Fig. 8).

**Figure 4:**
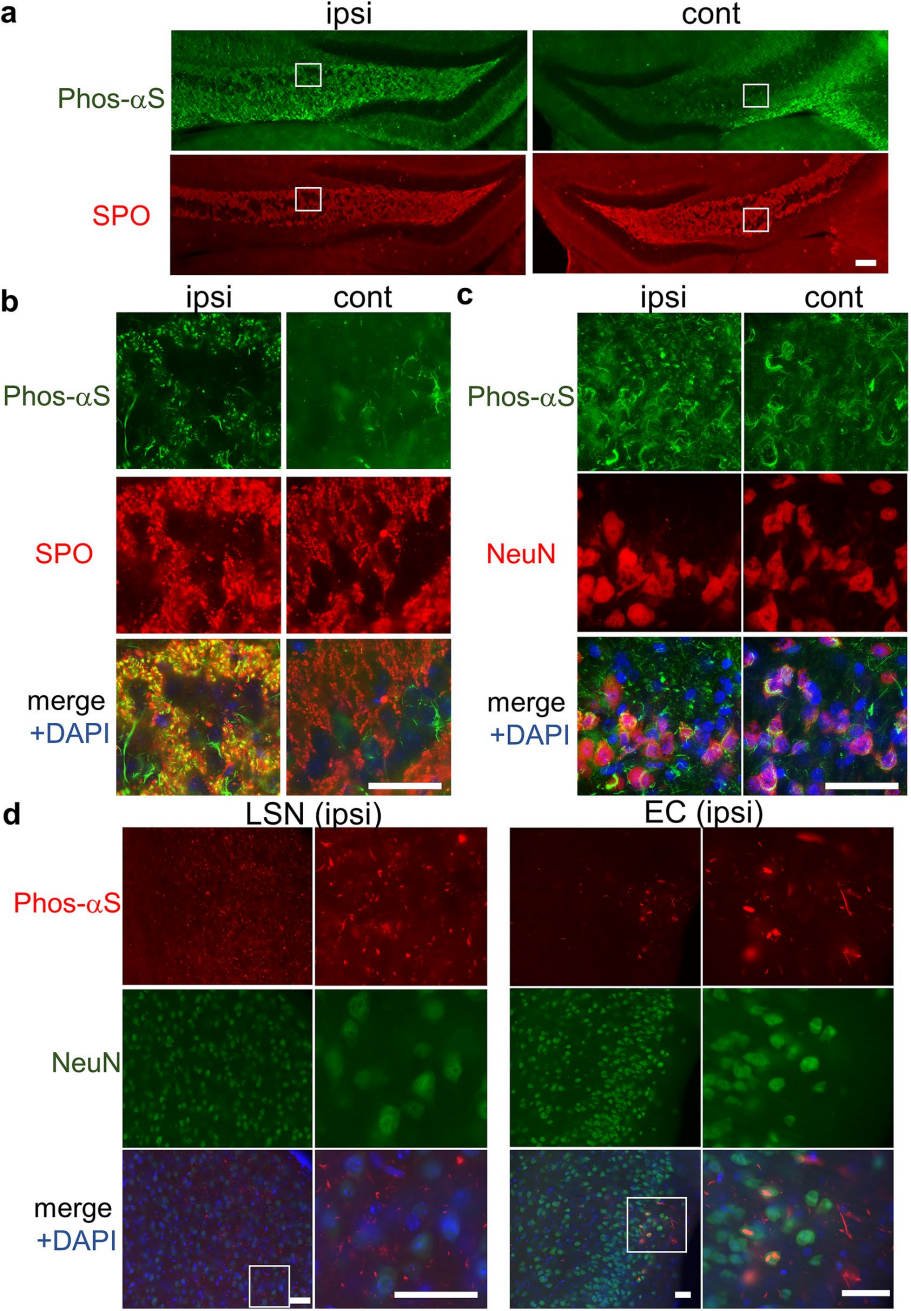
Difference in appearance of seed-dependent phosphorylated α-synuclein between the ipsilateral and contralateral sides. (**a**, **b**) Appearance of phosphorylated α-synuclein (Phos-αS) in the dentate gyrus (DG) and CA3 regions of the ipsilateral (ipsi, left) and contralateral (cont, right) sides. At 14 days after the injection, mice were sacrificed and the brains were fixed. Slices were double-stained with anti-phosphorylated α-synuclein and anti synaptoporin (SPO) antibodies. Images show phosphorylated α-synuclein (upper, green) and synaptoporin (lower, red). Magnified images of the regions surrounded by white rectangles are shown in b. The merged images with DAPI (blue) are also shown. (**c**) Slices were double-stained with anti-phosphorylated α-synuclein and anti NeuN antibodies. Images show phosphorylated α-synuclein (green), NeuN (red), and the merged image with DAPI (blue). (**d**) Appearance of phosphorylated α-synuclein in the lateral septal nucleus (LSN, left) and the entorhinal cortex (EC, right) regions. Images show phosphorylated α-synuclein (red), NeuN (green), and the merged image with DAPI (blue). Regions surrounded by white rectangles in merged images are magnified and shown in the rightmost panel of each lane. Scale bars, 100 μm (**a**), 50 μm (**b**, **c** and **d**).

At 14 days after the injection into mouse brain, phosphorylated α-synuclein was accumulated on the contralateral side as well as on the injected side (Fig. 4a). There were many phosphorylated dot-shaped α-synuclein accumulations in the dentate gyrus (DG) and CA3c region on the injected side and these dot-shaped accumulations were co-localized with synaptoporin, which is particularly enriched in mossy fiber synapses in the hippocampus^35^. We could not find phosphorylated dot-shaped α-synuclein accumulations positive for synaptoporin on the contralateral side (Fig. 4a, b). However, many NeuN-positive phosphorylated α-synuclein accumulations were observed in the CA3c region on the contralateral side (Fig. 4c). The CA3c region, into which α-synuclein PFFs were injected in our study, is known to receive input from the ipsilateral DG, CA3c and contralateral CA3c. The areas where large amounts of phosphorylated α-synuclein were observed are consistent with these regions, suggesting that phosphorylated α-synuclein was retrogradely transported from the CA3c region^36^. To verify this, we investigated the accumulations of phosphorylated α-synuclein in other regions that are neuroanatomically connected to the CA3c region. Accumulations of phosphorylated α-synuclein were also found in the lateral septal nucleus (LSN) and the entorhinal cortex (EC), supporting the idea that phosphorylated α-synuclein was retrogradely transported from the CA3c region (Fig. 4d)^36,37^. To examine whether the increase of phosphorylated α-synuclein continues also at late stages, we investigated the accumulation of phosphorylated α-synuclein in contralateral hippocampus at one month after the injection (Supplementary Fig. 9). We found that phosphorylated α-synuclein began to be accumulated in contralateral DG and the aggregates like forms of phosphorylated α-synuclein in contralateral CA3c seemed to be increased suggesting that both the amount and area of phosphorylated α-synuclein increase with time as previously reported^12,13,22^.

**Figure 5:**
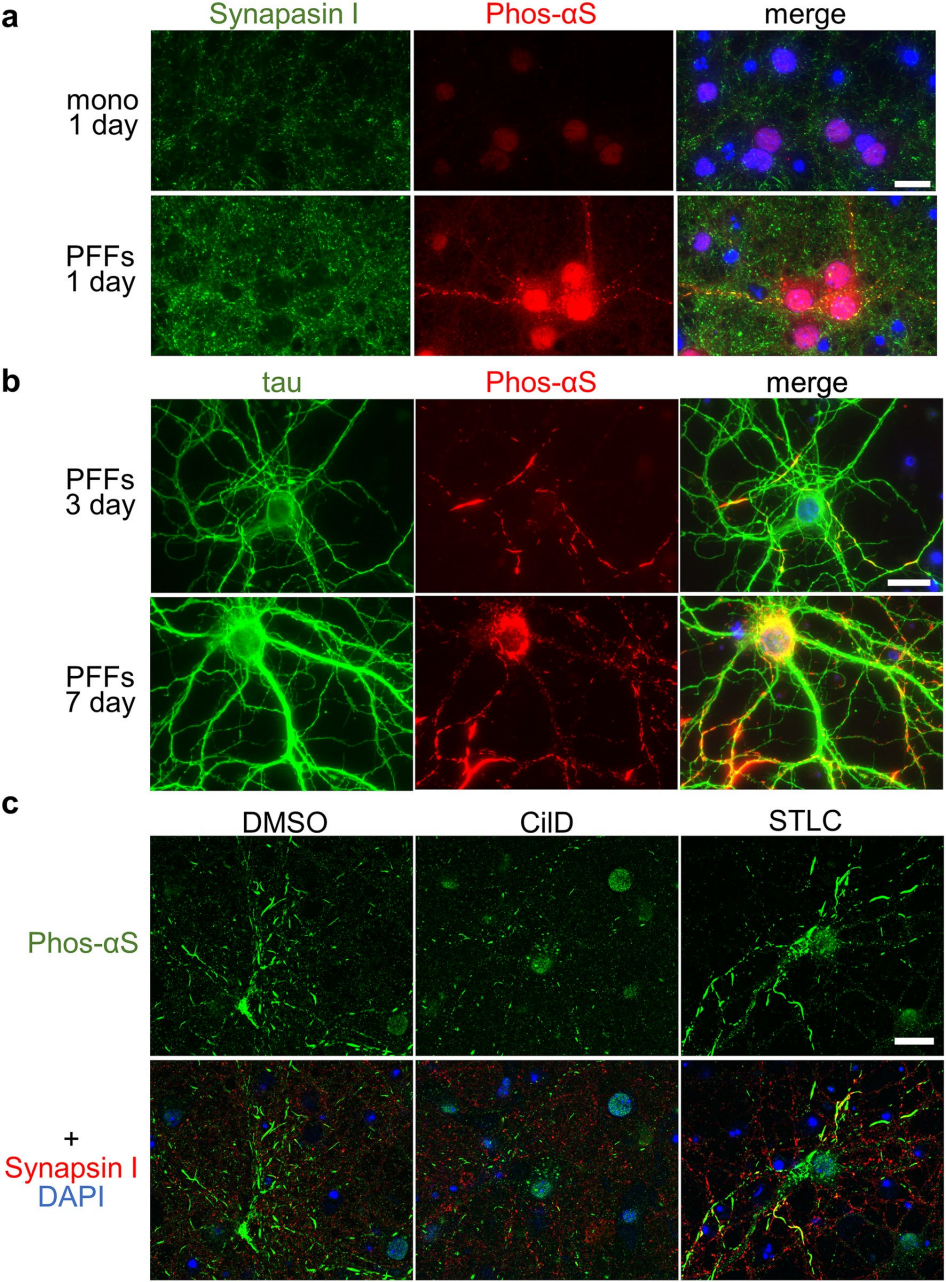
Seed-dependent phosphorylation of α-synuclein in pre-synaptic region of primary-cultured neurons. (**a**) Seed-dependent phosphorylation of α-synuclein (Phos-αS) in the pre-synaptic region. Primary-cultured cells grown in vitro for 14 days were treated with α-synuclein monomer (upper) or pre-formed fibrils (PFFs) (lower), and after one day, the cells were fixed and stained with anti-synapsin I (green) and anti-phosphorylated α-synuclein (red). Nuclei were stained with DAPI. Merged images are shown. (**b**) Seed-dependent phosphorylation of α-synuclein in axons. Primary-cultured cells grown in vitro for 14 days were treated with α-synuclein pre-formed fibrils (PFFs), and after 3 days (upper) or 7 days (lower), the cells were fixed and stained with anti-tau (green) and anti-phosphorylated α-synuclein (red). Nuclei were stained with DAPI. Merged images are shown. (**c**) Confocal laser microscopy analysis of cells treated with DMSO or drugs. Primary-cultured cells were treated with DMSO (left), CilD (center) or STLC (right) and stained with anti-phosphorylated α-synuclein (upper, green) and anti-synapsin I (lower, red). Nuclei were also stained with DAPI (lower, blue). Scale bars, 20 μm.

For confirmation, we performed similar experiments using primary-cultured mouse cells^38^. First, we examined whether α-synuclein is also expressed at presynaptic regions in primary-cultured cells from mouse brain^31^. We found little expression of synapsin I or α-synuclein at 7 days in vitro (DIV), whereas large amounts of α-synuclein were expressed in presynaptic regions at 14 DIV (Supplementary Fig. 10)^39^. We therefore introduced α-synuclein monomers or PFFs into primary-cultured mouse cells at 14 DIV and examined the appearance of phosphorylated α-synuclein the following day. We found that phosphorylated α-synuclein appeared only in the PFFs-transfected cells and was co-localized with synapsin I (Fig. 5a). These findings suggest that extracellular α-synuclein fibrils are taken up from presynaptic regions in primary neurons and induce the phosphorylation of intrinsic α-synuclein, which is abundant in presynaptic regions. Next, we examined the appearance of phosphorylated α-synuclein 3 or 7 days after fibril introduction. The phosphorylated α-synuclein appeared as processes like LNs at 3 days and as processes and aggregates like LBs at 7 days after introduction (Fig. 5b). We also examined the co-localization of tau and phosphorylated α-synuclein and found that phosphorylated α-synuclein mainly appeared in presynaptic regions and axons at 3 days and in axons and cell bodies at 7 days after introduction (Fig. 5b). As described above, in mouse brain injected with α-synuclein PFFs, phosphorylated α-synuclein forms aggregates in the cell body 14 days after the injection (Fig. 1b). These results suggest that phosphorylated α-synuclein is likely to be retrogradely transported from the presynaptic region through the axon both in primary-cultured cells and in mouse brain. To verify this, we used ciliobrevin D (CilD), a dynein inhibitor, and S-Trityl-L-cysteine (STLC), a kinesin inhibitor, because dynein and kinesin are known to be involved in retrograde and anterograde axonal transport, respectively^40–43^. Cells were treated with drugs at a day after PFFs introduction and fixed at two days after drug treatment. We found the cells treated with CilD had small phosphorylated α-synuclein processes while the cells treated with DMSO or STLC had large processes like LNs (Fig. 5c). Taken together, these results suggested that when extracellular α-synuclein fibrils (seeds) are initially taken up in presynaptic regions, intrinsic α-synuclein, which is abundant there, is phosphorylated in a seed-dependent manner and then retrogradely transported through the axon to the cell body, where it forms large aggregates.

## Discussion

In previous models employing the injection of PFFs into the mouse brain, the pathology has usually been evaluated several months after the injection^12,13,33,44^. In this study, we focused on the initial phase after the injection, and sought to determine how extracellular fibrils are taken up and how they seed intrinsic α-synuclein. Remarkably, we found that phosphorylated α-synuclein pathology appeared at presynaptic regions only 3 days after the injection. In agreement with previous findings, we found that endogenous α-synuclein is highly expressed at presynaptic regions in both mouse brain and primary-cultured neurons (Supplementary Fig. 1 and 10)^3,39,45^. The seed-dependent aggregate formation associated with prion-like propagation requires the presence of high concentrations of monomers, suggesting that presynaptically incorporated α-synuclein seeds efficiently induce intrinsic α-synuclein aggregation and phosphorylation^21,39^. If α-synuclein aggregate is taken up from the presynaptic regions, it is likely to be transported to the cell body by retrograde axonal transport^43^. This idea seems to be consistent with previous reports that extracellular α-synuclein is taken into the cell and transported to the cell body by the endolysosomal pathway, because endolysosomes are also thought to be transported from the synapse to the cell body by retrograde axonal transport^26,27,46^. In this study, time-course observations of injected mouse brain and primary-cultured cells revealed that the amount of phosphorylated α-synuclein increased concomitantly with retrogradely transport through the axon to the cell body. If the structure of intrinsic α-synuclein, which is abundant at the presynaptic region, is abnormally altered by seeds, then phosphorylated and transported to the cell body by the endolysosomal pathway along with other damaged proteins and organelles, it would be interesting to know the physiological role of the α-synuclein phosphorylation on the protein quality control.

On the other hand, the possibility that α-synuclein aggregates are taken up from sites other than presynaptic sites cannot be ruled out. Some aggregates may be taken up directly into the cell body by endocytosis and other aggregates may be taken up from the postsynaptic region. When phosphorylated α-synuclein is used as a marker of abnormal pathology, as in the present study, the incorporated aggregates must act as seeds, and seed-dependent conversion of normal α-synuclein into abnormal form must occur. Free α-synuclein is required for seed-dependent aggregation, but free α-synuclein is abundant in presynaptic regions^5^. It will be necessary to analyze the uptake of extracellular α-synuclein aggregates via other pathways and to assess their function as seeds by other methods, such as the use of fluorescently labeled fibrils or microfluidic chambers which separate synaptic terminals from cell bodies.

Previous studies have favored the idea that α-synuclein phosphorylation occurs after the formation of LBs or LNs^3,47^. However, in this study we found that α-synuclein is already phosphorylated in the presynaptic regions. Notably, there are reports of phosphorylated α-synuclein beginning to accumulate in presynaptic regions and axons in patients’ brains^48–50^. It is unclear whether this is the case in living patients, but if it is, it would be physiologically significant, because if the abnormal structure of α-synuclein induced by the seed is already phosphorylated in presynaptic regions, the kinase responsible for α-synuclein phosphorylation is likely to be localized at these regions. As a next step, it will be important to investigate the fate of α-synuclein transported to the cell body, including degradation by proteasomes or autophagy, aggregate formation, or release by endocytosis.

In this study, we injected higher amount of α-synuclein PFFs into the mouse brain than has been the case in the past^12,13,28^, in order to observe pathological induction of phosphorylated α-synuclein at an earlier stage. If the amount of α-synuclein PFFs to be injected is above a certain threshold, we think that the timing of the initiation of α-synuclein phosphorylation will not change much depending on the amount of α-synuclein to be injected. It has been reported that phosphorylated α-synuclein appears as early as one week after the injection, even with a much smaller amount of PFFs injection than ours^51^. On the other hand, we have reported that the amount of phosphorylated α-synuclein pathology increases with increasing injection volume^52^. When injecting a large amount of α-synuclein and/or observing the induction of pathogenesis early after the injection, it may be necessary to distinguish between the injected PFFs and the induced pathology, such as the use of S129A PFFs or antibodies specific for intrinsic α-synuclein.

Here, we show the spread of retrograde phosphorylated α-synuclein in neurons, but it is not clear whether phosphorylated α-synuclein itself is neurotoxic. In the brain of α-synucleinopathies patients or animal models, it has been reported that neurodegenerations were followed by retrograde degeneration of axons^53,54^. It would be interesting to show that the retrograde propagation of phosphorylated α-synuclein that we have reported here is related to retrograde neurodegeneration by examination of neuroinflammation. In fact, we examined the induction of inflammation, which is thought to be related to neurodegeneration, by Iba1 staining in the early stage after PFFs injection, but unfortunately, the expression of Iba1 was increased even when α-synuclein monomer was injected, and no obvious differences were observed between monomer and PFFs injected mice. In the future, detailed analysis of the induction of inflammation, synaptic terminal abnormalities, axonal degeneration or neurodegeneration in the α-synuclein PFFs introduced mice or cells and clarification of the cytotoxicity of α-synuclein aggregates and/or phosphorylated α-synuclein will lead to clarification of the pathogenesis of α-synucleinopathies and development of therapeutic methods.

It is thought that tau and TDP-43, as well as α-synuclein, propagate through the brain in a prion-like manner^5,55–57^. As described above, α-synuclein is abundant in presynaptic regions, whereas tau is abundant in neurites and TDP-43 is mostly localized in the nucleus. Consistent with previous reports, we found in the present study that localization and expression levels are important for prion-like propagation^39^. If TDP-43 aggregates are taken up from the presynaptic region like α-synuclein, they would need to be transported to the nucleus or cell body by retrograde axonal transport to act as seeds. In addition, the expression of TDP-43 in neurons is not likely to be as high as that of α-synuclein^32^. These considerations may explain why prion-like propagation of TDP-43 is less commonly observed in WT mice than that of α-synuclein.

If many neurodegenerative diseases are caused by aggregates spreading in the brain through prion-like propagation, aggregate uptake or seed-dependent aggregation could be an attractive target for disease treatment. If α-synuclein seeds are taken up at presynaptic regions, as we have shown here, and seed-dependent aggregation occurs at these regions soon afterwards, these steps could be good targets for treatment or prevention for neurodegenerative diseases. The accumulation of phosphorylated α-synuclein occurred in a very short period of time in the mouse and cellular models that we used in this study, and so these models might be helpful for drug screening of therapeutics specific to this step. We believe our findings will be useful in studies aimed at the treatment of neurodegenerative diseases involving accumulation of protein aggregates.

## Methods

### Animals

Mice (C57BL/6J for injection experiments and Jcl:ICR for primary-cultured cells) were obtained from Japan SLC. All experimental protocols were performed according to the recommendations of the Animal Care and Use Committee of Tokyo Metropolitan Institute of Medical Science, and all animal experiments were carried out in accordance with ARRIVE guidelines (The ARRIVE guidelines 2.0). All experimental protocols were approved by the Animal Care and Use Committee of Tokyo Metropolitan Institute of Medical Science (approval number 21-037).

### Expression and purification of recombinant WT and S129A mutant mouse α-synuclein

WT and S129A mutant mouse α-synuclein encoded in pRK172 plasmids were transformed into *E. coli* BL21 (DE3). Recombinant proteins were purified as described previously^44^. Protein concentration was determined by HPLC.

### Preparation of α-synuclein fibrils

α-Synuclein fibrils were prepared as follows^44^. Purified recombinant α-synuclein proteins were dissolved in 30 mM Tris–HCl, pH 7.5 and 0.1% NaN_3_ to a final concentration of 6 mg/ml. The samples were incubated at 37 °C under shaking for 7 days. The assembled α-synuclein was centrifuged at 113,000 g for 20 min, and the pellets were washed with saline and centrifuged again. The pellets were resuspended in saline. To determine the concentration of insoluble α-synuclein, proteins were disaggregated with 6 M guanidine hydrochloride and subjected to HPLC. α-Synuclein fibrils were sonicated for 30 seconds twice at 30% intensity using a Sonifier SFX250 cup horn sonicator (BRANSON) before use.

### α-Synuclein injection into mice

Six-week-old male C57BL/6J mice were purchased from Japan SLC. α-Synuclein samples (10 mg/ml, 5 μl) were injected into the hippocampus *Cornu Ammunis* 3c (CA3c) region (anterior–posterior, -2.0 mm; medial–lateral, 1.5 mm; dorsal–ventral, -2.0 mm from the bregma and dura). Injection into mouse brain was performed as described previously^13^. Monomer-injected WT mice were used as negative controls. At 3, 5 or 14 days after the injection, mice were deeply anesthetized with isoflurane (Pfizer) and sacrificed, and the brain was perfused with saline. Sections were fixed in 10% formalin neutral buffer solution (Wako).

### Primary-cultured cells and introduction of α-synuclein proteins into cells

Dissociated cultures of embryonic (E15) mouse cortical cells were prepared from pregnant Jcl:ICR mice using Neuron Dissociation Solutions (FUJI FILM Wako) according to the manufacturer’s protocol. Briefly, dissected brain was digested with enzyme solution for 30 min at 37 °C, then centrifuged. Dispersion solution was added and tissues were suspended, then isolation solution was added. Cells were collected by centrifugation, resuspended and plated on poly-L-lysine-coated cover glasses. Cells were maintained at 37 °C in 5% CO_2_ in Neurobasal Medium (Gibco) supplemented with 1 × B27 (Gibco) and 1 × Glutamax (Gibco). They were cultured for 7 or 14 days in vitro (DIV) in 6-well plates, and then treated with sonicated α-synuclein fibrils diluted in culture medium. Cells were collected or fixed at 1, 3 or 7 days post treatment. For the inhibition of the axonal transport, cells were treated with 20 μM CilD (Merck Millipore) or 20 μM STLC (Merck Millipore) as previous reports^41,42^. Two days after the drug treatments, cells were fixed and stained.

### Antibodies

Antibodies used in the present study were as follows. Anti-phospho-α-synuclein antibodies against pSer129 were 81A (Bio Legend) and EP1536Y (abcam). An anti-α-synuclein antibody was Syn202 (Santa Cruz). Anti-NeuN (abcam), anti-synapsin I (abcam), anti-tau (tauC, in house), anti-synaptoporin (Synaptic Syatems), anti-MAP2 (abcam), anti-Neurofilament L (Cell Signaling Technology), anti-inoinized calcium-binding adapter molecule 1 (Iba1) (Wako), anti-glial fibrillary acidic protein (GFAP) (abcam) or anti-2',3'-Cyclic-nucleotide 3'-phosphodiesterase (CNPase) (Cell Signaling Technology) were also used. Alexa488- or Alexa568-conjugated secondary antibodies (Thermo) were used.

### Immunofluorescence staining and FSB staining of mouse brains

Fixed brains were sectioned at 30-µm thickness using a VT1200 vibratome (Leica). Free-floating sections were mounted on glass slides and processed for antigen retrieval by heating at 105 °C in 0.01 M sodium citrate buffer, pH 6.0, for 1 min. Sections were then permeabilized with 0.5% Triton X-100 in phosphate-buffered saline and blocked with Blocking One Histo (Nacalai tesque). The sections were treated with primary antibodies diluted in PBST at room temperature overnight. After washing with PBST, the sections were treated with Alexa488- or Alexa568-conjugated secondary antibodies (1:1000; Thermo) for 1 h. After washing, the sections were coverslipped using Vectashield with DAPI (Vector). Images were acquired on a BZ-X710 fluorescence microscope (Keyence) and analyzed with BZ-X analyzer (Keyence). The colocalization coefficient was calculated by Image J. For FSB staining, the sections treated with first and second antibodies were stained with 0.001% FSB (Dojindo) for 30 min and washed twice with 50% ethanol. Images were acquired on a LSM780 confocal microscope (Zeiss).

### Immunocytochemistry

Introduction of α-synuclein fibrils was conducted as described above, using mouse primary-cultured cells grown on coverslips. Cells were fixed with 4% paraformaldehyde, permeabilized with 0.5% Triton X-100 in phosphate-buffered saline and blocked with Blocking One Histo. The cells were treated with the primary antibodies for 1 h, then washed and treated with secondary antibodies conjugated with Alexa Fluor for 1 h. The cells were mounted using Vectashield with DAPI to counterstain nuclear DNA and analyzed with a BZ-X710 (Keyence) and BZ-X analyzer (Keyence). To avoid the fluorescence of drugs, images of the cells treated with drugs were acquired on a LSM780 confocal microscope.

### Statistical analysis

Statistical analysis of data was carried out using R version 3 (The R Foundation for Statistical Computing). Three independent experiments were carried out and the colocalization coefficient was calculated by Image J. The differences between means of two groups were analyzed using one-way ANOVA and Tukey post hoc test. *P* values below 0.05 were considered to be statistically significant. The data are mean ± standard error of mean (S.E.M).

## Supplementary Information

Supplementary Information.

## Data Availability

The data that support the findings of this study are available either within the body of the manuscript, within the supplementary information files, or are available from the corresponding author upon reasonable request.
