# Supplementary material for: Phosphorylation of endogenous α-synuclein induced by extracellular seeds initiates at the pre-synaptic region and spreads to the cell body
Source: Sci Rep. 2022 Jan 21;12:1163. doi: 10.1038/s41598-022-04780-4 (PMC8782830; doi:10.1038/s41598-022-04780-4)
Supplement: Supplementary file 1 — Supplementary Information. [file 41598_2022_4780_MOESM1_ESM.pdf]

Supplementary Information

Phosphorylation of endogenous α-synuclein induced by extracellular seeds initiates at the pre-synaptic region and spreads to the cell body

Shiori Awa[1,2†#], Genjiro Suzuki[1†*], Masami Masuda-Suzukake[1], Takashi Nonaka[1], Minoru Saito[2,3] and Masato Hasegawa[1*]

[1] Department of Brain and Neuroscience, Tokyo Metropolitan Institute of Medical Science, Tokyo, Japan. [2]Department of Biosciences, College of Humanities and Sciences, Nihon University, Tokyo, Japan. [3] Department of Correlative Study in Physics and Chemistry, Graduate School of Integrated Basic Sciences, Nihon University, Tokyo, Japan

#Current address: Graduate School of Frontier Biosciences, Osaka University, Osaka, Japan

[†]These authors contributed equally to this work

*Corresponding authors: suzuki-gj@igakuken.or.jp, hasegawa-ms@igakuken.or.jp

TEL: +81-3-6834-2349

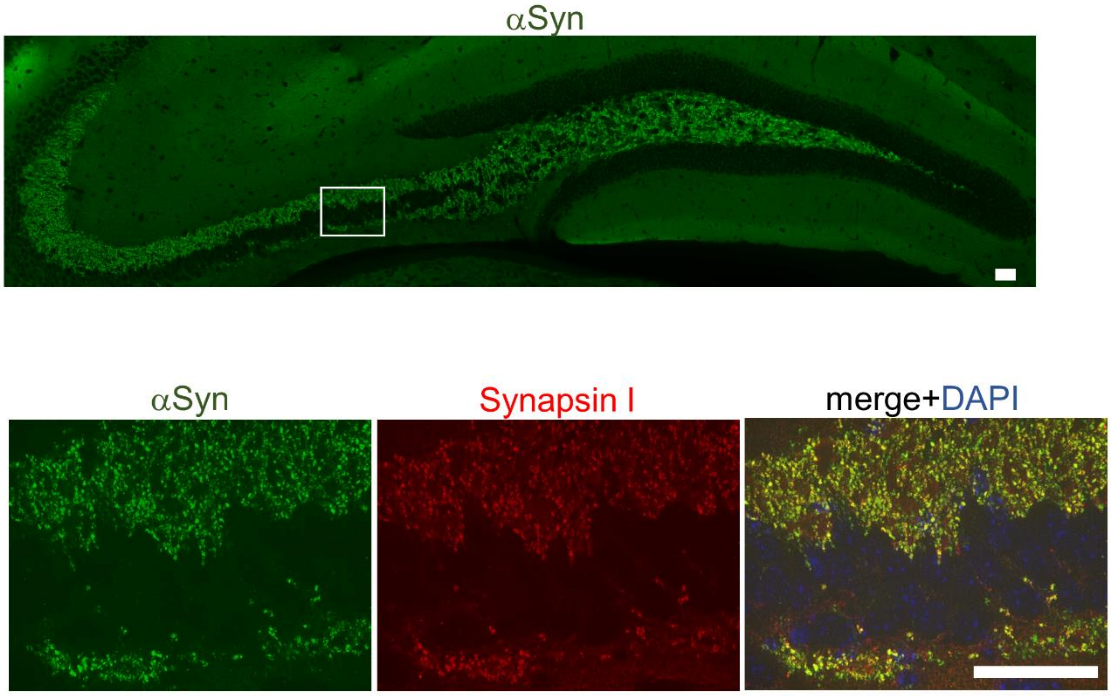

Supplementary Figure 1. Expression of α-synuclein in the hippocampal presynaptic region.

Slices of WT mouse hippocampal region were double-stained with anti-α-synuclein (α-Syn) antibodies (green) and anti-synapsin I antibodies (red). Nuclei were stained with DAPI. Magnified images of regions surrounded by white rectangles are shown in the lower panels. Scale bars, 50 μm.

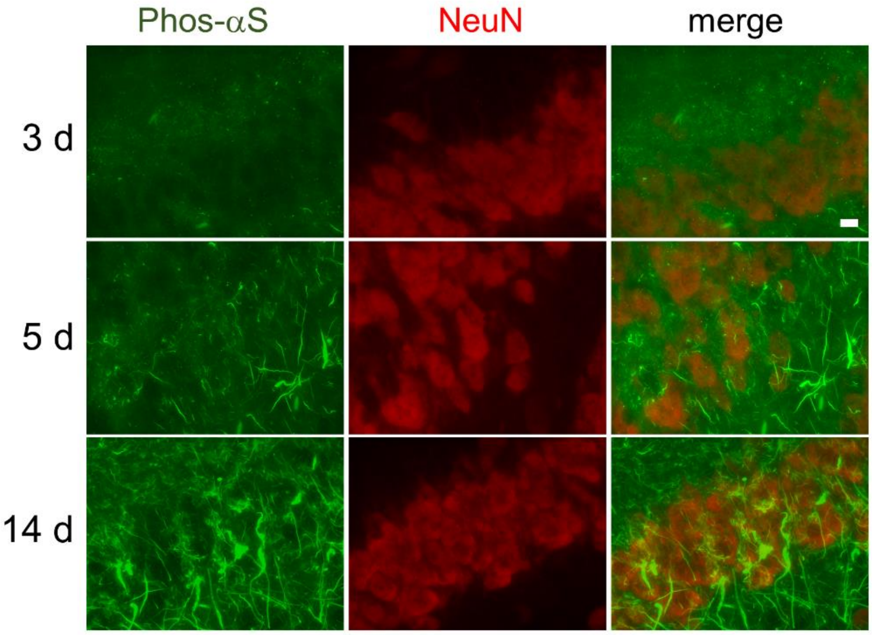

Supplementary Figure 2. Seed-dependent phosphorylation of α-synuclein in mouse brain.

Mouse WT α-synuclein PFFs were injected into the CA3c region of mouse hippocampus. At 3 days (upper), 5 days (middle) or 14 days (lower) after the injection, mice were sacrificed, and the brains were fixed. Images show phosphorylated α-synuclein (Phos-αS, green), NeuN (red), a neuronal cell body marker, and the merged image (right) in the CA3c region. Scale bars, 10 μm.

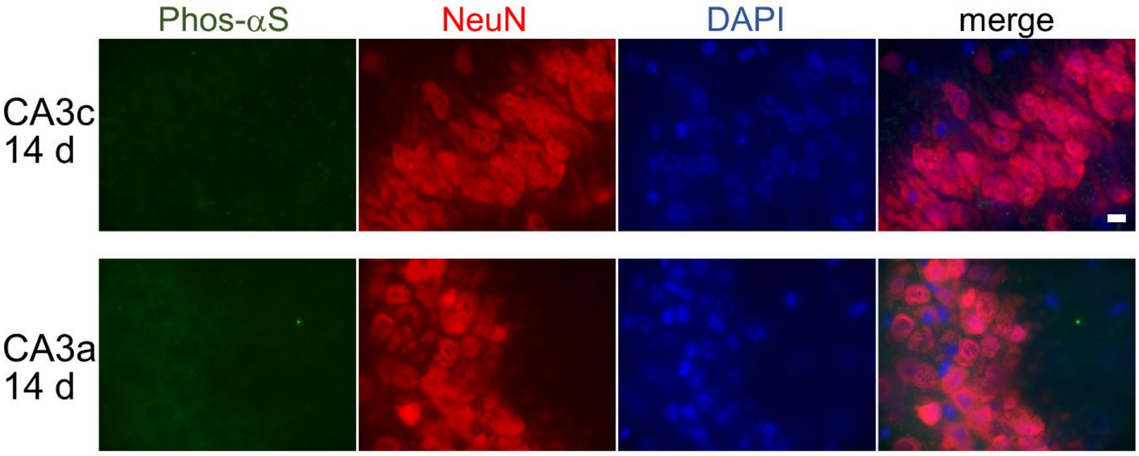

Supplementary Figure 3. Negative controls for seed-dependent phosphorylation of α-synuclein in mouse brain.

Mouse S129A α-synuclein monomer was injected into the CA3c region of mouse hippocampus. At 14 days after the injection, mice were sacrificed, and the brains were fixed. Images show phosphorylated α-synuclein (Phos-αS, green), NeuN (red), a neuronal cell body marker, nuclei stained with DAPI (blue) and the merged image (right) in the CA3c region (upper) and CA3a region (lower). Scale bars, 10 μm.

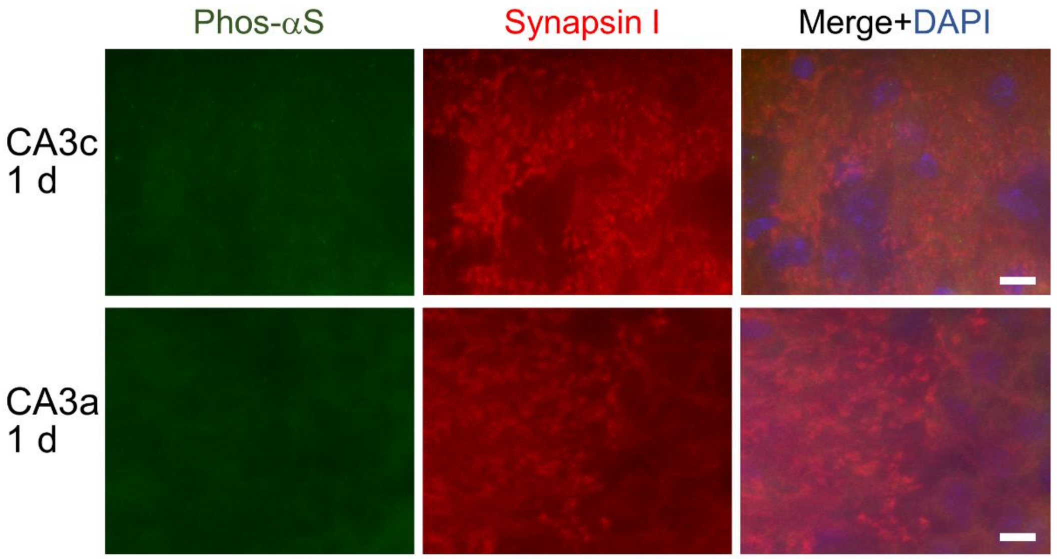

Supplementary Figure 4. Phosphorylated α-synuclein signal at one day after the injection.

Mouse S129A α-synuclein fibrils were injected into the CA3c region of mouse hippocampus. At one day after the injection, mice were sacrificed, and the brains were fixed. Images show phosphorylated α-synuclein (Phos-αS, green), Synapsin I (red), nuclei stained with DAPI and the merged image (right) in the CA3c region (upper) and CA3a region (lower). Scale bars, 10 μm.

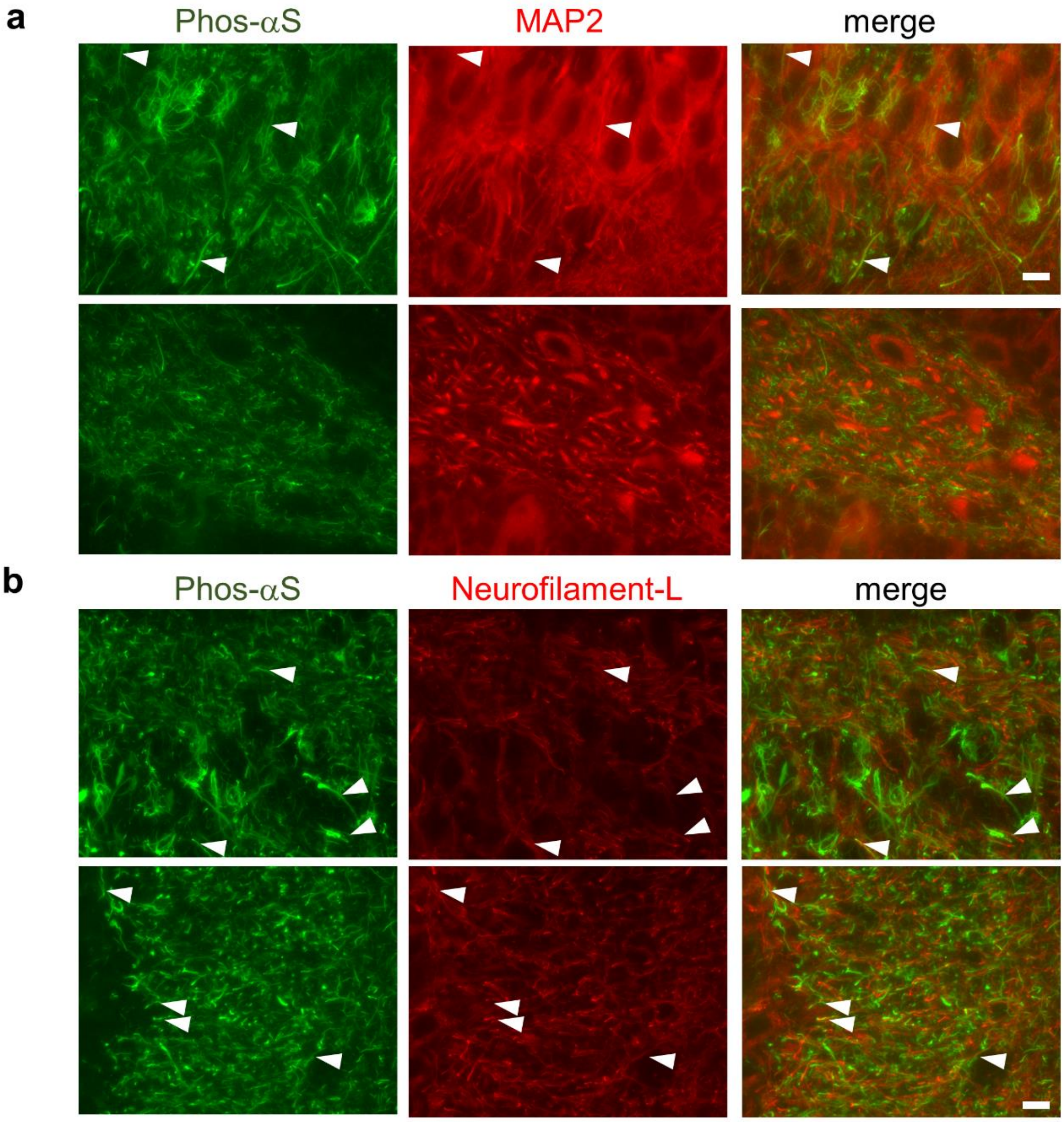

Supplementary Figure 5. Double-stained images of phosphorylated α-synuclein and neuronal markers.

At 14 days after the injection, mice were sacrificed, and the brains were fixed. **a** Images show phosphorylated α-synuclein (Phos-αS, green), MAP2 (red) and the merged image in the CA3c region (upper) and CA3a region (lower). **b** Images show phosphorylated α-synuclein (green),

neurofilament-L (red) and the merged image in the CA3c region (upper) and CA3a region (lower).

White arrow heads indicate the colocalization of phosphorylated α-synuclein with these markers.

Scale bar, 10 μm.

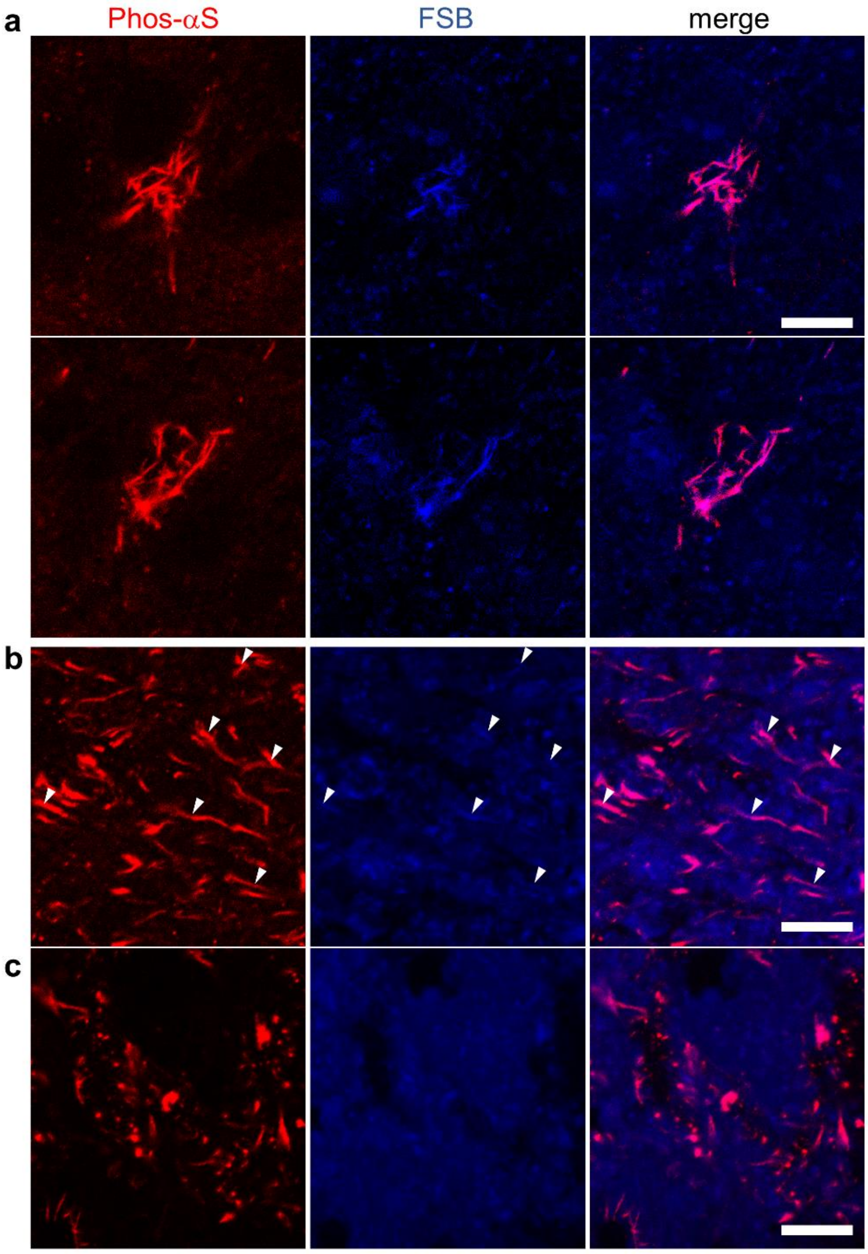

Supplementary Figure 6. Double-stained images of phosphorylated α-synuclein and FSB.

At 14 days after the injection, mice were sacrificed and the brains were fixed. Images show phosphorylated α-synuclein (Phos-αS, red) and FSB (blue). **a** Aggregate-like structures found in the CA3c regions. **b** Process-like structures found in the CA3a regions. Arrow heads indicate FSB positive structures. **c** Dot-like structures found in the CA3c regions. Scale bars, 20 μm.

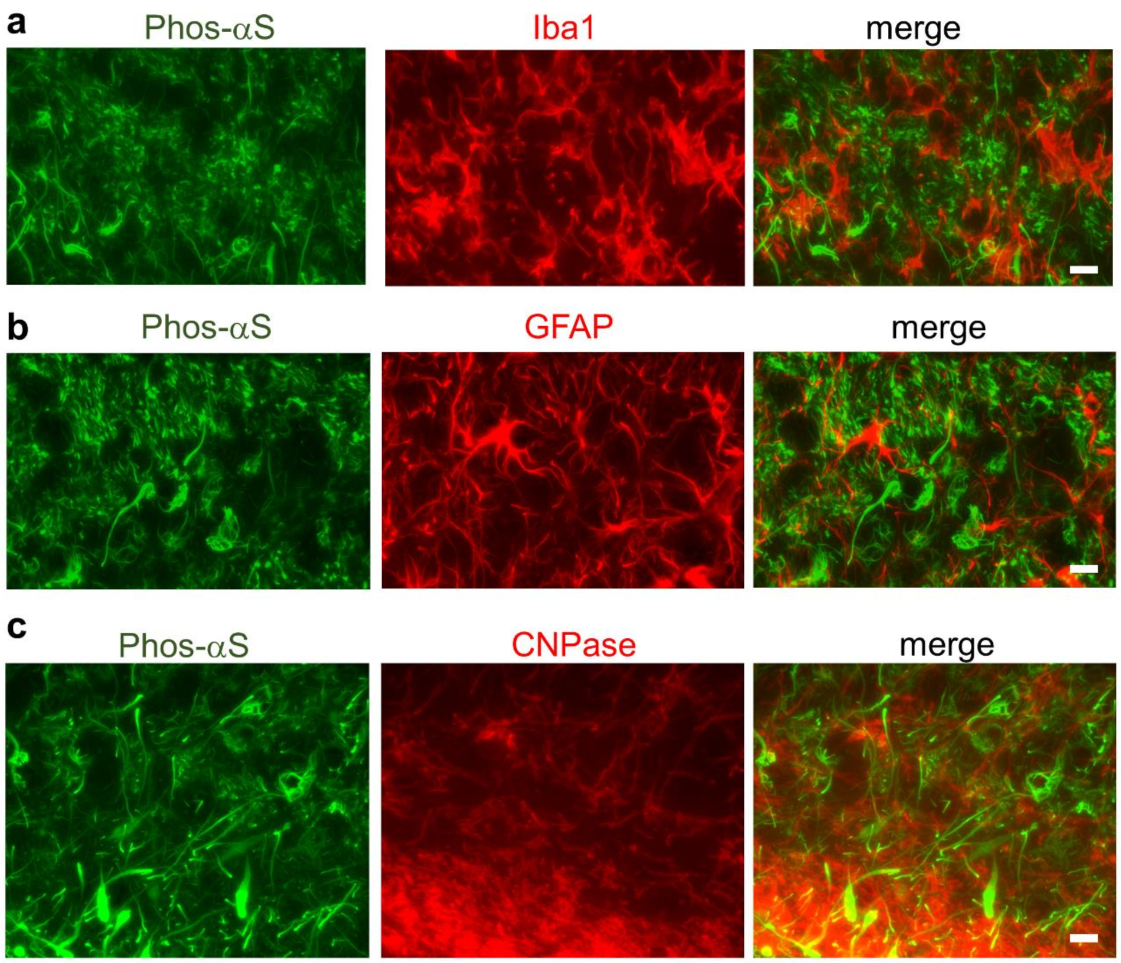

Supplementary Figure 7. Double-stained images of phosphorylated α-synuclein and glial markers. At 14 days after the injection, mice were sacrificed and the brains were fixed. **a** Images show phosphorylated α-synuclein (Phos-αS, green), Iba1 (red) and the merged image in the CA3c region. **b** Images show phosphorylated α-synuclein (green), GFAP (red) and the merged image in the CA3c region. **c** Images show phosphorylated α-synuclein (green), CNPase (red) and the merged image in the CA3c region (upper) and DG region (lower). Scale bar, 10 μm.

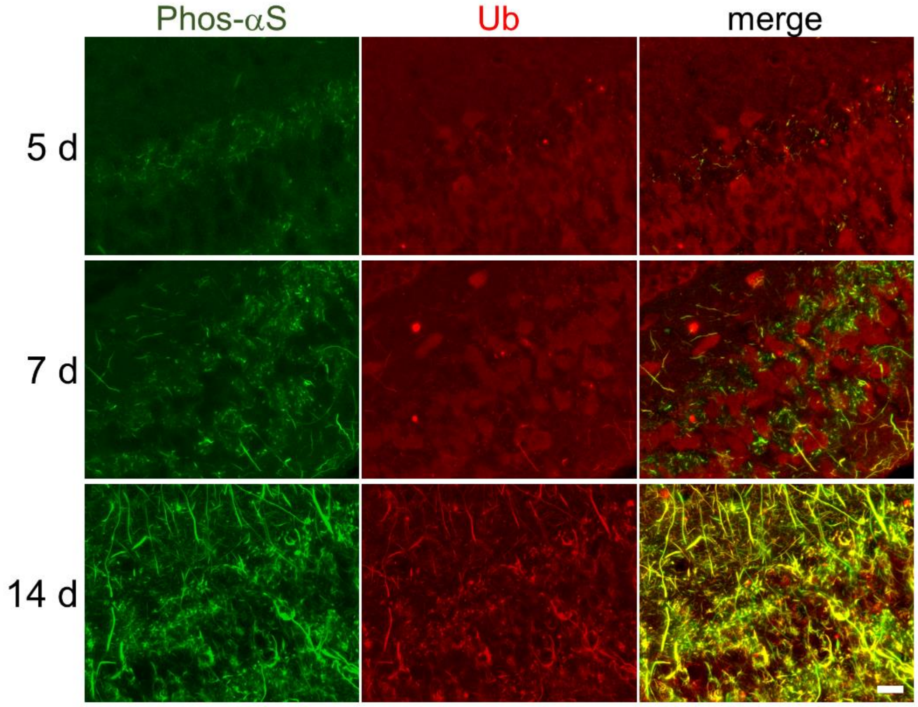

Supplementary Figure 8. Double-stained images of phosphorylated α-synuclein and ubiquitin. At 5 days (upper), 7 days (middle) or 14 days (lower) after the injection, mice were sacrificed and the brains were fixed. Images show phosphorylated α-synuclein (Phos-αS, green), ubiquitin (Ub, red), and the merged image (right) in the CA3c region. Scale bar, 20 μm.

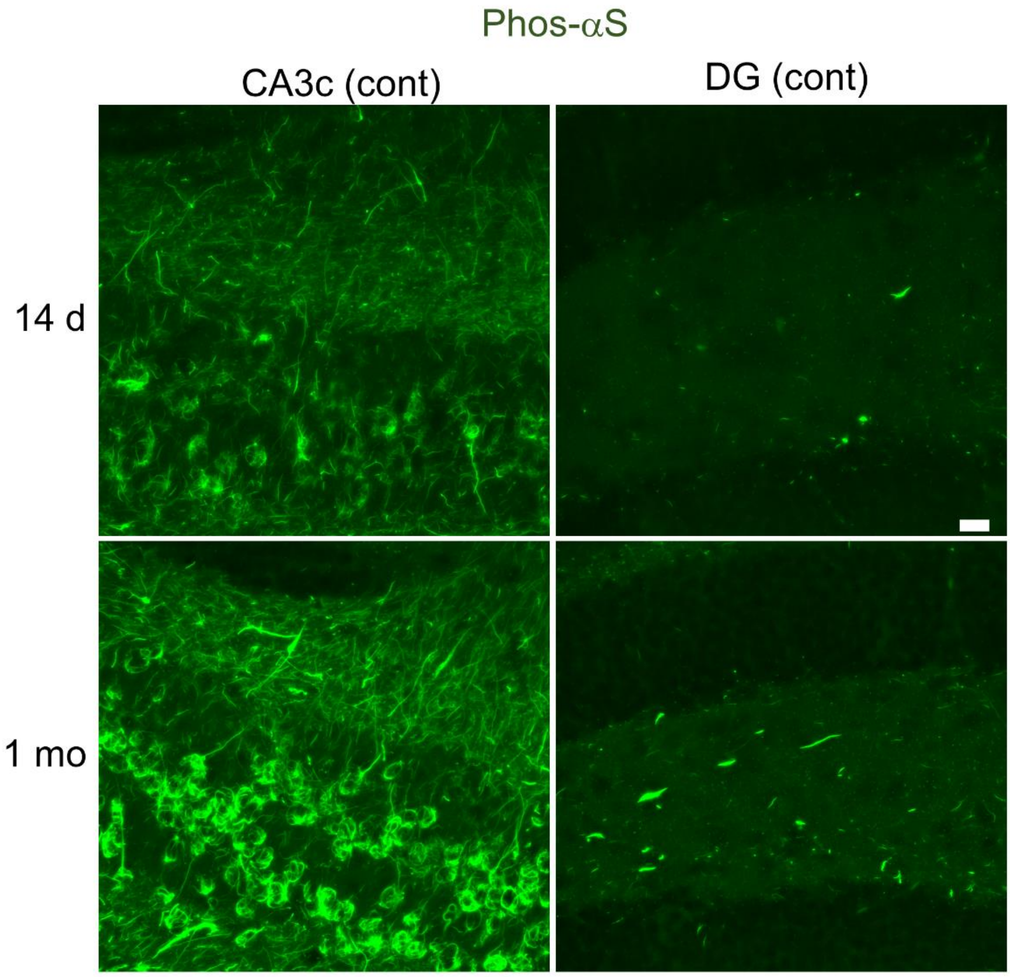

Supplementary Figure 9. Appearance of phosphorylated α-synuclein at one month after the injection. At 14 days (upper) or one month (lower) after the injection, mice were sacrificed and the brains were fixed. Images show phosphorylated α-synuclein (Phos-αS, green) in the contralateral CA3c regions (left) or contralateral DG regions (right). Scale bar, 20 μm.

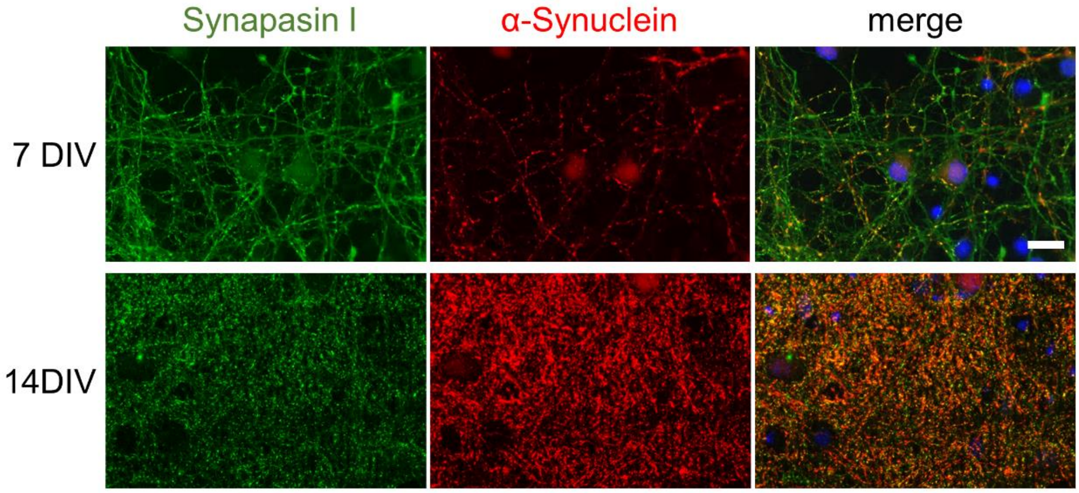

Supplementary Figure 10. Expression of α-synuclein in the pre-synaptic region in primary-cultured neurons. Expression of synapsin I (green), a pre-synaptic marker, and α-synuclein (red) in primary-cultured neurons grown in vitro for 7 days (upper) and 14 days (lower). Scale bar, 20 μm.
